# Mediterranean Diet, Telomere Maintenance and Health Status among Elderly

**DOI:** 10.1371/journal.pone.0062781

**Published:** 2013-04-30

**Authors:** Virginia Boccardi, Antonietta Esposito, Maria Rosaria Rizzo, Raffaele Marfella, Michelangela Barbieri, Giuseppe Paolisso

**Affiliations:** Department of Internal Medicine, Surgical, Neurological Metabolic Disease and Geriatric Medicine, Second University of Naples, Naples, Italy; University College London, United Kingdom

## Abstract

Leukocyte telomere length (LTL) and rate of telomere shortening are known biomarkers of aging while, numerous studies showed that Mediterranean diet (MD) may boost longevity. We studied association between telomere length, telomerase activity and different adherence to MD and its effects on healthy status. The study was conducted in 217 elderly subjects stratified according Mediterranean diet score (MDS) in low adherence (MDS≤3), medium adherence (MDS 4–5) and high adherence (MDS≥6) groups. LTL was measured by quantitative polymerase chain reaction and telomerase activity by a PCR-ELISA protocol. High adherence group showed longer LTL (p = 0.003) and higher telomerase activity (p = 0.013) compared to others. Linear regression analysis including age, gender, smoking habit and MDS showed that MDS was independently associated with LTL (p = 0.024) and telomerase activity levels (p = 0.006). Telomerase activity was independently associated with LTL (p = 0.007) and negatively modulated by inflammation and oxidative stress. Indeed, telomerase levels were associated with healthy status independently of multiple covariates (p = 0.048). These results support a novel role of MD in promoting health-span suggesting that telomere maintenance, rather than LTL variability is the major determinant of healthy status among elderly.

## Introduction

Telomere length, or more precisely, the rate of telomere shortening, is a biomarker of biological aging [1] and eating nutrient-rich foods might delay aging process and reduces risk of many chronic diseases [2]. The role of telomere length in cellular senescence and development of chronic disease associated with physiological aging has been addressed in several studies [3], [4]. Although telomere length may predict clinical outcomes and mortality among humans, cells with shortened telomeres remain genetically stable if the telomere maintenance system, which includes mainly telomerase, is fully functioning [5]. Metabolic factors, such as abdominal fat and increased circulating glucose levels are related to shorter telomeres and lower telomerase activity [6]–[8], supporting the role of lifestyle and environmental factors on telomeres maintenance. The effect of diets on human health has already been evaluated in many studies while limited are evidences on the relative importance of dietary intake on telomeres maintenance and stability. During the past years population-based surveys and large-scale clinical trials have provided scientific evidences that diet, and especially those rich in fruits, vegetables, fish and low-fat dairy products, are associated with lower incidence of various chronic diseases and higher survival [9], [10]. Accordingly longer LTL is related to a more healthy diet, including greater intake of antioxidant [11], [12], less processed meat consumption [12], intake of fruits and vegetables and less dietary fat [13], [14]. Various nutrients as well as changes in diet and lifestyle have been already shown to influence telomere length through mechanisms reflecting their role in cellular functions including inflammation, oxidative stress, DNA integrity and DNA methylation [2].

The Mediterranean diet (MD) is one of the healthiest dietary pattern in the world due to its relation with low morbidity and mortality for some chronic diseases [15], [16]. This diet has benefits on risk factors for cardiovascular diseases such as lipoprotein levels, endothelium vasodilatation, insulin resistance, antioxidant capacity, incidence of acute myocardial infarction, and global cardiovascular mortality [15]. Increasing evidences show that adherence to Mediterranean-diet style correlates to higher longevity and healthy aging, not only in countries from the Mediterranean Basin but also in other non-Mediterranean countries as well [17], [18]. A possible link between telomere length or maintenance and MD has been also recently suggested. A more recent *in vitro* study showed that Mediterranean diet protects the cells from oxidative stress preventing cellular senescence, cellular apoptosis and reducing telomere attrition [19]. Whether a different adherence to MD may affect telomeres length and/or PBMC telomerase activity is still unknown and poorly investigated. Thus, we aim at investigating the hypothesis that the lower adherence to MD pattern might be associated with markers of accelerated cellular aging, including reduced telomerase activity and shortened telomere length in a cohort of elderly subjects from the South Italy. Considering that PBMC telomerase activity is associated with a lower incidence of age-related diseases [20], we also aim at investigating the potential association between telomere length, telomerase activity and Mediterranean diet adherence as well as their effects on healthy status among elderly subjects.

## Materials and Methods

### Ethics Statement

Investigation has been conducted in accordance with the ethical standards. After a clear explanation of the potential risk of the study, all subjects provided written informed consent to participate in the study, which was approved by the Ethical Committee of the Second University of Naples.

### Study Population

385 Caucasians subjects living in Campania (Southern Italy) and referred to our Department have been screened. To avoid bias, individuals with evidence of acute inflammatory or infectious diseases, diabetes, malignancies, immunologic or hematologic disorders or treatment with anti-inflammatory drugs were excluded from the study. In total 217 subjects have been selected. At enrolment a dietary questionnaires to participants was administered and a full medical history was collected. Clinical information was obtained by routine laboratory analyses, history and physical examination. Data collection included an interview concerning demographics, health-related behaviors, functional status and cognitive function. A validated health questionnaire [21] was used to determine the presence, history of, or absence of the following diseases: hypertension, congestive heart failure, myocardial infarction, peripheral vascular disease, dementia, cancer, stroke, chronic obstructive pulmonary disease (COPD), and diabetes. We used the Barthel Activities of Daily Living (ADL) Index, a validated instrument, to assess physical function (scores range from 0 to 100) [22]. “Healthy” old were defined as those individuals with a Barthel score >90 (independent range) and the absence of all of the following diseases: hypertension, congestive heart failure, myocardial infarction, peripheral vascular disease, dementia, stroke, and COPD. “Unhealthy” old had Barthel scores <80 (requiring at least some assistance) and two or more of the above diseases [23].

### Mediterranean Diet Score

The degree of adherence to the traditional MD was assessed using the Mediterranean Diet Score (MDS) according to the method developed by Trichopoulou et al [24]. Intake of each of 9 food groups was dichotomized using sex-specific median values as cut-offs. A score of 1 was assigned for above the median level of presumed beneficial foods (vegetables, legumes, fruits, cereal, fish and ratio of monounsaturated fats to saturated fats) and consumptions below the median level of presumed detrimental foods (meat and dairy products). For ethanol, 1 point was assigned to men who consumed between 10 and 50 g per day and to women who consumed between 5 and 25 g per day. Thus, the total MDS ranged from 0 (minimal adherence to the traditional Mediterranean diet) to 9 (maximal adherence). For analytical purposes, we categorized the MDS into three groups as follows: low adherence (MDS≤3), medium adherence (MDS 4–5) and high adherence (MDS≥6) to the diet [24]. Subjects showing inconstant adherence to diet in the last three months were ruled out.

### Analytical Methods

Anthropometric determinations (weight, height and body mass index, BMI) were measured by standard techniques. Blood samples were collected in the morning after the participants had been fasting for at least 8 hours. Plasma glucose was determined immediately by the glucose oxidase method (Glucose Autoanalyzer, Beckman Coulter, Inc., Fullerton, CA, USA). Plasma fasting cholesterol and triglycerides were determined by routine laboratory methods (Roche Diagnostics, GmbH, Mannheim, Germany). Serum concentrations of IL-6 were determined in duplicate using a highly sensitive, quantitative sandwich enzyme assay (Quantikine HS PharmPak, R&D Systems). High-sensitivity TNF-a was assayed by immunonephelometry on a Behring Nephelometer 2 (Dade Behring, Marburg, Germany). Plasma C-reactive protein was determined using automated turbidimetry. Nitrotyrosine plasma concentration, marker of oxidative stress, was assayed by enzyme linked immunosorbent assay. Nitrotyrosine was determined because this modified amino acid is a product of free-radical (O^2–^) interaction with nitric oxide (NO). The interaction of O^2–^ with NO is very rapid and leads to inactivation of NO and production of the potent oxidant peroxynitrite. Detection of nitrotyrosine is strongly suggestive of increased generation of peroxynitrite [25].

### Telomerase Activity Measurement in PBMC

PBMC were isolated by centrifugation in a Lympho-Ficoll gradient and preserved at −80°C before analysis. Telomerase activity in PBMC was measured using a commercial telomerase PCR-ELISA (Roche Diagnostics Corp., Indianapolis, IN, USA), based on the telomeric repeat amplification protocol. The assay procedures followed the recommendations of the manufacturer, with each sample being analyzed in triplicate.

### LTL Measurement

Mean telomere length was measured quantitatively in genomic DNA from white blood cells. Genomic DNA was prepared using a commercial DNA extraction kit following the manufacturer’s instructions (Nucleic Acid and Protein Purification-Macherey-Nagel). Average TL in peripheral white blood cells was measured using a validated quantitative polymerase chain reaction (Q-PCR) method as described by Cawthon [26] which measures the relative average TL in genomic DNA by determining the ratio of telomere repeat copy number to single-copy gene copy number (*T*/*S* ratio) in experimental samples relative to a reference sample. Telomere primers were tel1b, 5′-cggtttgtttgggtttgggtttgggtttgggtttgggtt-3′, final concentration 100 nM; and tel2b, 5′-ggcttgccttacccttacccttacccttacccttaccct-3′, final concentration 900 nM. Single copy gene (β-globin) primers were hbg1, 5′-gcttctg acacaactgtgttcactagc-3, final concentration 300 nM; and hbg2, 5′-caccaacttcatccacgttcacc-3′, final concentration 700 nM. The *T* signal for an experimental DNA sample is the number of nanograms of the reference DNA that matches the experimental sample for copy number of the telomere repeats. All samples were measured in triplicate, and the average was used for analyses. The coefficient of variation was 1.98%. Results obtained using this method correlate very well with those obtained with the traditional terminal restriction fragment (TRF) length by Southern blot technique [26]. To obtain the TL for the reference DNA, we used the *T*/*S* ratios of 30 DNA samples with known mean TRF lengths obtained by Southern blot technique. The relative T/S ratios and the means telomere length by the Southern blot approach significantly correlated (r = 0.667; p = 0.001). The slope of the linear regression line through a plot of *T*/*S* ratio (the *x* axis) versus mean TRF length (the *y* axis) is the number of base pairs of telomeric DNA corresponding to a single *T*/*S* unit. TL was successfully measured in all 217 individuals.

### Calculations and Statistical Analyses

The observed data are normally distributed (Shapiro-Wilk W-Test) and presented as means ± Standard Deviation (SD). One-way ANOVA followed by Bonferroni multiple testing correction was used to assess differences in clinical and biochemical data among the presented groups. Pearson correlation coefficients were calculated to evaluate the relationship between subjects age and the LTL as indicated.

A cluster analysis allowed us to evaluate whether clustering of variables of inflammation was associated with LTL variability. For this purpose, we created a compound score, referred to as a clustering score. A z score quantifies the original score in terms of the number of SDs that score is from the mean of the distribution. It was calculated as the sum of the z scores of the main variable of inflammation (IL-6, TNF-α and CRP). A z score indicates the position of an individual value of a variable in the total distribution of the variable in the population and is calculated as follows: (individual value - mean value)/SD.

Linear regression analysis was used to test the association of the different adherence to Mediterranean Diet with LTL and telomerase activity levels independently of multiple confounding factors. A binary logistic regression analysis was used to test the association between telomerase activity levels and healthy status independently of multiple covariates. Because some of the predictor variables used in the regression analyses were correlated, we performed collinearity analysis. Using variance inflation factors (VIF), no collinearity problems were detected in our data. Sample size calculation was estimated on an IBM PC computer by GPOWER software. The resulting total sample size, estimated according to a global effect size of 25% with type I error of 0.05 and a power of 90% was 207 patients. All *p* values presented are 2-tailed and a *p*≤0.05 was chosen for levels of significance. Statistical analyses were performed using SPSS 17 software package (SPSS, Inc., Chicago, IL) or GraphPad Prism software version 5.0 (San Diego, CA,USA).

## Results

### Characteristics of all Study Population

217 unrelated Caucasians subjects (115 (53%) men and 102 (47%) women) were enrolled to the present study. Participants were old (mean age = 77.9±2.7 years) and slightly overweight (Body Mass Index (BMI) = 25.8±1.4 kg/m^2^) with a similar proportion between men and woman. The mean age of participants was 78.0±2.9 years for men (range 71–86) and 77.7±2.5 years for women (range 71–87). According to gender ratio women had significantly lower BMI (25.3±1.3 kg/m^2^ vs 26.2±1.4 kg/m^2^, p = 0.001) compared men, while no differences among other clinical and biochemical characteristics as well as inflammatory and oxidative stress biomarkers were found (data not shown).

As expected in the all study population a statistically significant inverse correlation between LTL and age (r = −0.258, p<0.001) was found. In particular, each 1-y increment in age, LTL decreases by 0.058 Kb. Independently of age, women had significantly longer LTL than men (5.06 Kb±0.56 Kb and 4.73 Kb±0.60 Kb respectively, p<0.001), while no difference in PBMC telomerase activity levels (0.88±0.20 OD vs 0.88±0.19 OD) between gender was found.

Stratifying subjects in non smoker, former smoker and current smoker, a statistically significant difference in LTL variability between groups was found (4.98 Kb±0.61 Kb, 4.92 Kb±0.54 Kb and 4.49 Kb±0.53 Kb respectively, p<0.001), while no difference in basal telomerase activity between groups was found (0.89±0.20 OD, 0.85±0.21 OD and 0.91±0.17 respectively, p = 0.375 ).

**Figure 1:**
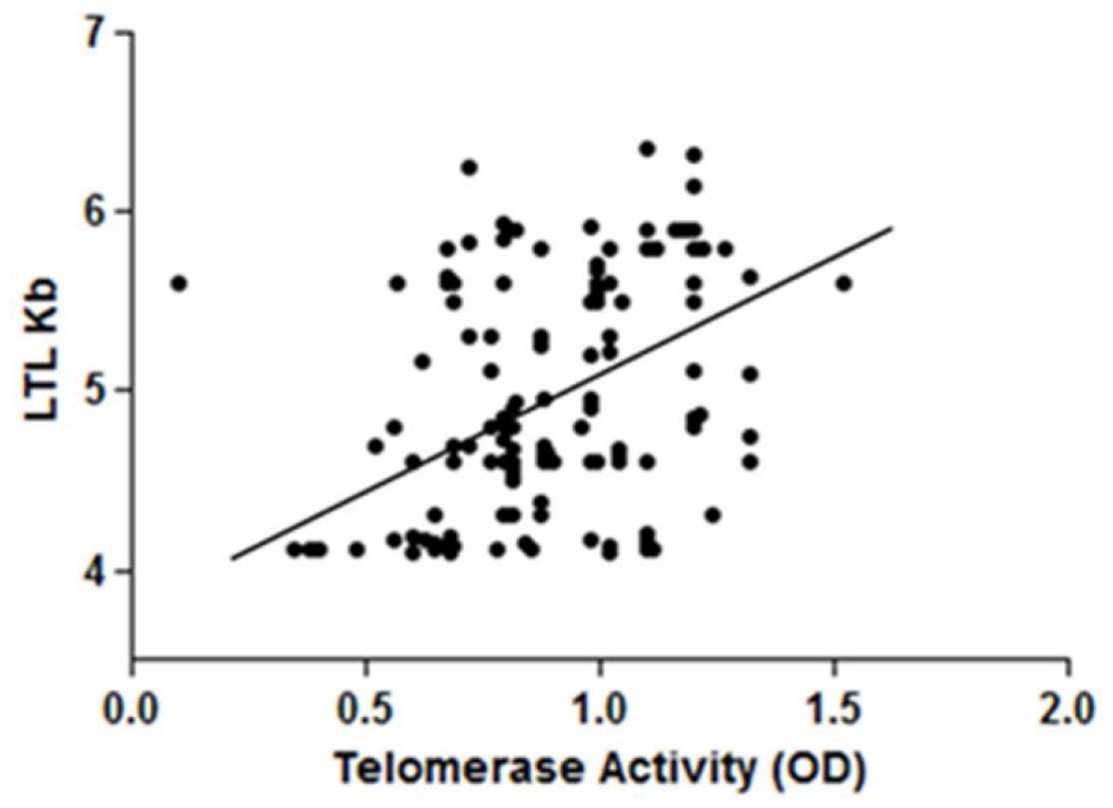
Correlation between LTL and telomerase activity in all population study. Partial correlations (r = 0.208; p = 0.002) between Leukocyte Telomere Length (LTL) and PBMC telomerase activity in all study population (n = 217), adjusted by gender, age and smoking habit.

A significant positive correlation (r = 0.151; p = 0.028) between telomere length and PBMC telomerase activity levels was found even after adjustment for age, gender and smoking habit (Figure 1).

### Characteristics of Study Population according to Mediterranean Diet Score

Table 1 describes the general clinical and biochemical characteristics of participants according to MDS (Mediterranean Diet Score) subgroups: low adherence (MDS≤3), medium adherence (MDS 4–5) and high adherence (MDS≥6). 32.3% showed low adherence (MDS≤3) to the Mediterranean diet, 31.3% medium adherence (MDS 4–5) and 36.4% high adherence (MDS≥6) to this diet. No participants obtained a score of 9, as an indicator of the maximal adherence to the Mediterranean Diet. Higher frequency of female, no smokers and “healthy” subjects in the high adherence group were found.

**Table 1:** Characteristics of study population (n = 217) according to Mediterranean Diet Score.

	Mediterranean Diet Score			
	≤3(n = 70)	4–5(n = 68)	≥6(n = 79)	p
Age (years)	77.6±3.0	78.4±2.7	77.7±2.4	0.212
Gender (F/M)	30/40	26/42	46/33	0.034*
Smoking Status				
non smoker % (n)	24.0 (30)	37.6 (47)	38.4 (48)	0.009
former smoker % (n)	36.2 (21)	31.0 (17)	32.8 (20)	
current smoker % (n)	52.9 (19)	11.8 (4)	35.3 (11)	
Healthy % (n)	34.3 (24)	47.1 (32)	54.4 (43)	0.046*

Data are means ± standard deviation.

*p* values were obtained using χ^2^ test* or ANOVA.

**Figure 2:**
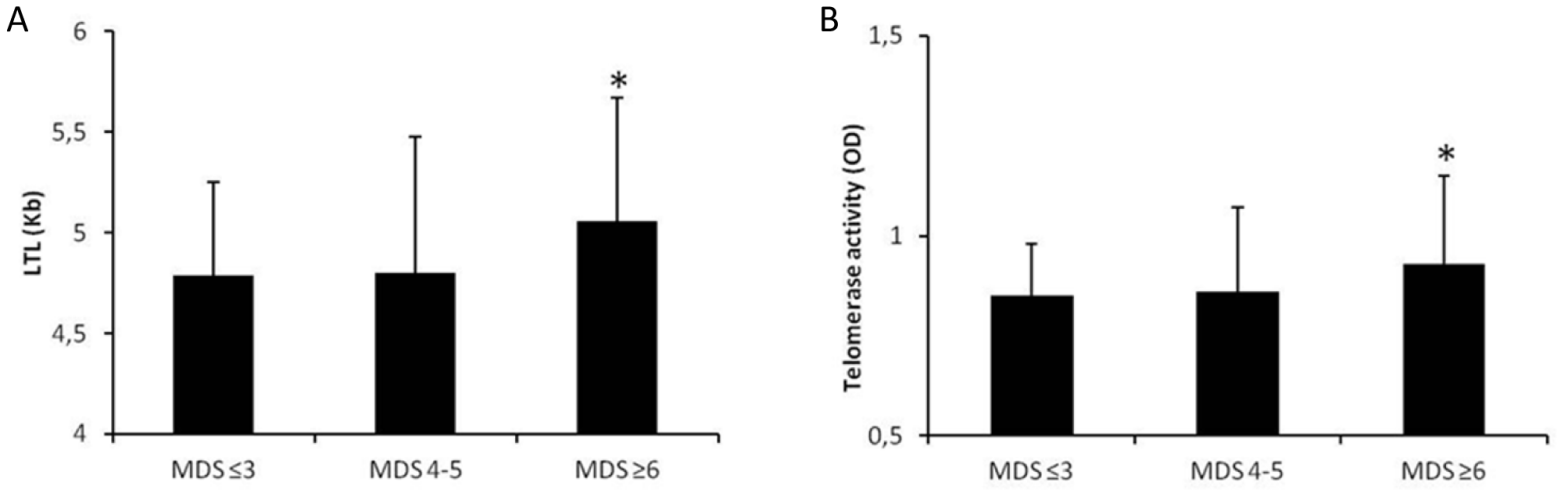
LTL and Telomerase Activity according Mediterranean Diet Score subgroups. Data are means ± standard deviation. MDS≤3: n = 70; MDS 4–5: n = 68; MDS≥6: n = 79. *p* values were obtained using ANOVA followed by Bonferroni multiple testing correction. A) *p = 0.003; MDS≥6 vs MDS≤3 p = 0.009 and MDS≥6 vs MDS 4–5 p = 0.011 B) *p = 0.013; MDS≥6 vs MDS≤3 p = 0.028 and MDS≥6 vs MDS 4–5 p = 0.033. LTL, Leukocyte Telomere Length; MDS, Mediterranean Diet Score.

Participants with the highest adherence to MD (MDS≥6) showed longer telomere length (p = 0.003) as well as higher telomerase activity (p = 0.013) compared to other groups (Figure 2). Such a difference resulted significant even after adjustment for age, gender and smoking habits (p = 0.043 and p = 0.048, respectively). Every 1-y increment in age, LTL decreases by 0.072 Kb (β = −0.456, p* = *0.001), 0.057 (β = −0.284, p* = *0.043) and 0.051 (β = −0.293, p* = *0.015) in low adherence, medium and high adherence groups respectively, showing a statistical different telomere attrition between MDS groups (p = 0.001) along with aging.

The independent effect of diet on telomere length and PBMC telomerase activity variability was tested by a linear regression analysis controlling by multiple covariates. Models including age, gender, smoking habit and MDS showed that MDS were independently associated with both LTL variability (β = 0.141 p = 0.024; R^2^ = 0.209) and telomerase activity levels (Table 2, Model 1). A model having LTL as dependent variable and age, gender, smoking habit, MDS and telomerase activity as independent variables, showed that telomerase activity (β = 0.167 p = 0.007; R^2^ = 0.226) but not MDS (β = 0.110 p = 0.181) was independently associated with LTL variability. In this latter model age, gender and smoking habit were independently associated with LTL variability.

**Table 2:** Linear regression analyses with telomerase activity as the dependent variable (n = 217).

Model 1	β	*p*	*R^2^*
Age	−0.050	0.468	
Gender	0.029	0.673	
Smoking habit	−0.050	0.397	
MDS	0.190	.0006	0.237
**Model 2**			
Age	−0.048	0.480	
Gender	0.024	0.720	
Smoking habit	−0.058	0.401	
MDS	0.107	0.188	
Inflammation score	−0.166	0.021	0.245

MDS = Mediterranean Diet Score calculated as low = 1, medium = 2, high = 3.

Gender calculated as female = 0, male = 1.

Smoking habit calculated as non smoker = 0, former smoker = 1, current smoker = 2.

### Mediterranean Diet, Inflammation and Telomere Maintenance System

Subjects with the highest adherence score (MDS ≥6) showed lower plasmatic levels of CRP (0.41±0.31 mg/dl vs 0.56±0.46 mg/dl, p = 0.018), IL-6 (2.27±0.76 pg/ml vs 2.55±0.65 pg/ml, p = 0.010), TNF-α (2.33±0.71 pg/ml vs 2.62±0.62 pg/ml, p = 0.021) and nitrotyrosine (0.23±0.07 µmol/L vs 0.26±0.07 µmol/L, p = 0.009) compared to low adherence group respectively.

A partial correlation analysis, controlled by age, gender and smoking habit, showed that LTL negatively correlated with inflammation score (r = −0.244; p<0.001) as well as with nitrotyrosine (r = −0.174; p = 0.011). PBMC telomerase activity levels negatively correlated with both inflammation (r = −0.138; p = 0.048) and nitrotyrosine levels (r = −0.157; p = 0.022). Inflammation score, calculated as the sum of the z scores of the main variable of inflammation, CRP, IL-6 and TNF-α, positively correlated with nitrotyrosine levels (r = 0.556, p<0.001).

Testing the effect of multiple covariates on telomerase activity levels, a linear regression analysis controlling by age, gender, smoking habit, MDS and inflammation score showed that inflammation but not MDS was independently associated with telomerase activity variability (Table 2, Model 2).

### Mediterranean Diet, Telomere Maintenance System and Healthy Status

Categorizing individuals as “healthy old” (Barthel score >90 and the absence of all of the following disease: hypertension, myocardial infarction, vascular diseases, dementia, stroke, congestive heart failure) and as “unhealthy old” (Barthel score <80 and two or more of the above disease), higher frequency of healthy subjects in the MD highest adherence group was found. Healthy old (n = 99) showed significantly longer telomere length (5.01 Kb±0.58 Kb vs 4.79 Kb±0.61 Kb, p = 0.008) compared the unhealthy group (n = 118) as well as higher PBMC telomerase activity (0.92±0.19 vs 0.85±0.20, p = 0.022). A binary logistic regression analysis with healthy status as dependent variable and age, gender, smoking habit, inflammation, telomerase activity levels, LTL and MDS as covariates showed that only telomerase activity was independently associated with a better health status (Odds ratio = 4.257, 95% CI = 1.011–17.926, p = 0.048).

## Discussion

Telomere attrition occurs as the result of cellular replication and is accelerated by various environmental factors such as inflammation and oxidative stress [1]. Longer LTL relates to a more healthy diet [27] even if, to the best of our knowledge, the relative effect of specific diet on LTL and telomerase activity in humans is poorly investigated [12], [14]. Mediterranean diet is one of the most healthy dietary pattern in the world due to its relation with a low morbidity and mortality for some chronic diseases, potentially affecting longevity and health-span [15], [16]. However, exactly how the traditional MD may affect life expectancy remains unknown, although evidences show that the overall dietary pattern and not any particular food may boost longevity. It is well established that one important effect of Mediterranean-style diet in prolonging lifespan is associated with reduced systemic oxidative stress and inflammation [28], [29]. Therefore, we hypothesized that Mediterranean diet would be associated with the rate of telomere shortening and telomerase activity in a manner consistent with reported associations between such diet and inflammation, chronic diseases, and rate of mortality.

Using data from a population-based study of elderly subjects from the Mediterranean area of Campania (South Italy), we found evidence of a retrospective association between high adherence to Mediterranean diet style and telomere maintenance system. The major findings of our investigation are: i) higher adherence to MD significantly correlates with telomere length, independently of multiple confounding variables affecting telomere attrition ii) PBMC telomerase activity is significantly and independently associated with LTL variability iii) MD is independently associated with LTL variability iiii) telomerase activity is associated with healthy status independently of multiple confounding factors, including LTL variability. So far, this is the first study aimed at investigating the relationship between telomere length, PBMC telomerase activity and adherence to Mediterranean diet among elderly people. Our study show that people with the highest adherence to this diet have longer telomere length and higher telomerase activity levels in peripheral white blood cells, independently of multiple confounding variables. We hypothesized that the high adherence to Mediterranean diet, influencing PBMC telomerase activity levels, is associated with lower telomere attrition along with aging. In fact, we found a positive association between telomere length and adherence to MD, independently of multiple confounding variables known to affect telomere attrition.

Many factors such as genetic and environmental factors modulate LTL attrition even if telomeres can remain genetically stable if the telomere maintenance system, which includes mainly telomerase, is fully functioning. Numerous evidences show that various nutrients as well as changes in diet and lifestyle may influence telomere length [14] through mechanisms which reflect their role in many cellular functions including inflammation, oxidative stress, and potentially PBMC telomerase activity [30]. Importantly, we found that longer LTL correlates with higher PBMC telomerase activity, while stratifying subjects according MDS, participants with higher adherence to MD show longer telomeres as well as higher PBMC telomerase activity. MDS is independently associated with both telomere length and telomerase activity levels, but most importantly the regression model show that the effect of MD on LTL variability is mediated by telomerase activity levels independently of multiple confounding factors. These findings are new and innovative supporting previous data showing that higher telomerase activity in PBMC in response to changes in diet and lifestyle [14], [30]. Recently, Ornish et al. [14] showed that diet can modulate telomerase activity in PMBC while if such an effect translated into change in the rate of telomeres shortening is unclear. Interestingly, here, we found that PBMC telomerase activity is significantly and independently associated with LTL variability. A potential explanation of such finding is that the high adherence to Mediterranean diet may stimulate PBMC telomerase activity, either directly by the effect of some specific nutrients including in the diet or indirectly by the global effect of diet on the modulation of inflammation and oxidative status. Accordingly we found that subjects with higher adherence to MD, have lower plasmatic level of inflammatory substrate and oxidative stress, as shown by lower levels of CRP, IL-6, TNF-α and nytrotirosine. The effect of diet is independent of the main variables affecting LTL variability. Interestingly, up-regulating telomerase in vitro promotes cell longevity and genomic stability [31] and no study evaluated such an effect *in vivo*. Telomerase activity is an important determinant of telomere length and may be considered a new marker measured in very few studies in human beings. According to previous studies [23], we show that elderly with a better healthy status have longer LTL, and we first show that the better healthy status is associated with higher PBMC telomerase activity. We tested the relationship between telomerase activity and healthy status and we found a significant positive association independently of multiple confounding factors, which suggests that telomere stability rather than LTL may be the major determinant of healthy status.

Thus, it is possible to speculate that the higher telomerase activity in PBMCs as a result of the quality of adherence to the MD, might have an important clinical relevance and may represent a new biomarkers of healthy aging. In recent studies conducted in mouse model, it has been show that telomerase activation inducted by gene therapy [32], [33] or TA-65 stimulator [34], delays physiological aging and extendslifespan. Most importantly these studies show that the activation of telomerase is associated to an improvement of certain health-span indicators including glucose tolerance, osteoporosis and skin fitness, without much increasing global cancer incidence. Again, in mouse, telomerase activity is associated to lower LDL cholesterol [14] playing an important role in the development of cardiovascular disease [35]. However, the significance of an increase in telomerase activity in humans remained to be elucidated. Our findings in an elderly population reveal a lower incidence of chronic disease such as hypertension, myocardial infarction, vascular diseases, dementia, stroke, congestive heart failure among people with higher circulating telomerase activity levels. For the first time, we show the effect among humans of Mediterranean diet on telomerase activity modulation. Our results, showing a higher frequency of healthy subjects with higher telomerase activity as well as longer telomeres in the high adherence diet subgroup, support a novel role of Mediterranean diet in promoting health-span. It is possible to speculate that the integrity of telomere maintenance and its related stability than individual LTL, is the major determinant of healthy status among elderly people.

The limited number of subjects and the lack of a replication study are potential limitations of this study. Another limitation is the method used to estimate mean telomere length, the qPCR assay. The assay has been reported to show a strong correlation with mean telomere length measured by the Southern blot [26]. In our study the correlation is slightly weaker. This finding could be explained by the small sample size and also because the Southern blot measures a variable amount of non-telomeric DNA, hence the correlation is not expected to be perfect. However, in our population study, we found a decrease in LTL with increasing aging as well as shorter LTL in men, which both represent a very good quality control. The quantitative fluorescence in situ hybridisation (qFISH) protocol [36], using specific acid probes to hybridize to the telomeric repeats would be more accurate compared with the used approach, thus, further studies will be necessary to replicate and validate our findings.

In conclusion our results give an evidence for an association between high adherence to the MD and a slower rate of cellular ageing. Taken together all these data support the novel hypothesis that a lower rate of telomere shortening and higher PBMC telomerase activity might be involved in lifespan and most importantly in health-span among populations consuming traditional Mediterranean diet.
